# Epigenetic Patterns in Musculoskeletal Disease: Methylation of *DKK1*, *RHOJ*, and *SOX6* Genes in Osteoarthritis and Osteoporosis

**DOI:** 10.3390/cimb48070644

**Published:** 2026-06-23

**Authors:** Anton V. Tyurin, Bulat I. Yalaev, Karina E. Akhiiarova, Ilmira I. Galina, Jie Li, Rita I. Khusainova

**Affiliations:** 1 Department of Internal Medicine, Bashkir State Medical University, Ufa 450000, Russia; liciadesu@gmail.com (K.E.A.); ilmira.ilfatovna@bk.ru (I.I.G.); khusainova.rita@endocrincentr.ru (R.I.K.); 2 Laboratory of Genomic Medicine, Endocrinology Research Center, Moscow 101000, Russia; yalaev.bulat@endocrincentr.ru; 3 School of Public Health, Southern Medical University, Guangzhou 510080, China; lijie4863@gdph.org.cn; 4 Global Health Research Center, Guangdong Provincial People’s Hospital, Guangzhou 510080, China

**Keywords:** osteoarthritis, osteoporosis, comorbidity, DNA methylation, *DKK1*, *SOX6*, *RHOJ*, epigenetics, pyrosequencing, postmenopausal women, biomarker

## Abstract

Osteoarthritis (OA) and osteoporosis (OP) are prevalent conditions with a complex relationship, yet their shared epigenetic mechanisms remain poorly understood. While genes like *DKK1*, *RHOJ*, and *SOX6* have been implicated in both diseases, the specific role of individual CpG sites has not been fully characterized. We investigated CpG methylation in these genes using bisulfite pyrosequencing of peripheral blood DNA from n = 96 postmenopausal women: n = 24 with comorbid OA and OP, n = 34 with OA, and n = 38 healthy controls. Methylation differences were analyzed using statistical tests and logistic regression. Comorbid patients showed significant hypermethylation at two *DKK1* CpG sites compared to the OA-only group (p_adj_ = 0.0007 and p_adj_ = 0.042). Conversely, one *DKK1* site was hypomethylated in the OA-only group relative to controls (p_adj_ = 0.03). A regression model combining three *DKK1* sites and one *SOX6* site demonstrated predictive value for comorbid disease, with an AUC of 0.696. These findings identify site-specific methylation of *DKK1* and *SOX6* as a molecular signature associated with comorbid OA and OP, offering new insights into their shared etiology.

## 1. Introduction

The increase in the incidence of osteoarthritis (OA), particularly of the knee and hip, as well as osteoporosis (OP), driven by the aging population, has led to a rise in healthcare expenditures [1]. The relationship between OA and OP has long been a subject of debate. High bone mineral density (BMD) has been implicated in the pathophysiology of OA [2], initially, OA and OP were considered mutually exclusive pathologies. To date, osteoporotic fractures are increasingly reported in patients with lower extremity OA, as well as the progression of OA in patients with OP [3]. Given the multifactorial nature of these diseases and the wide range of risk factors for both conditions, the search for common pathogenetic pathways, as well as potential molecular genetic predictors—as new tools for individual prognosis and potential highly selective therapeutic targets—is becoming increasingly relevant. According to the literature, associations with collagen genes (*COL1A1*) have been found for both OA and OP [4], and estrogen receptor genes [5] and vitamin D receptor genes [6], which play an important role in the pathogenesis of both diseases. In particular, genetic factors that influence BMD (e.g., components of the Wnt signaling pathway) also affect cartilage health [7]. This indicates common pathogenetic pathways between OA and OP [8]. However, the available data do not explain the full heterogeneity of both OA and OP [9], the mechanisms underlying the development of these pathologies, or the possible variants of OA and OP comorbidity, which necessitates a more in-depth and detailed investigation of the issue.

Epigenetics refers to heritable changes in gene and protein expression that occur independently of alterations in the DNA sequence and are mediated by modifications in chromatin state [10]. These modifications are reversible, highly dynamic, and respond to both endogenous signals and external stimuli. In recent years, epigenetic factors have been considered as biomarkers of aging and age-related diseases, which holds significant clinical relevance for diagnosis, prognosis, and prediction of therapeutic response. OP and OA are complex, multifactorial, late-onset disorders with a strong epigenetic component that differentially affect bone tissue metabolism [11], leading to an imbalance between bone formation by osteoblasts and bone resorption by osteoclasts. Recent studies examining global and gene-specific methylation profiles in whole blood have identified epigenetic signatures largely associated with the progression of OP and OA, which could represent non-invasive prognostic biomarkers [12]. However, the tissues involved in the pathogenesis of the two diseases are distinct: OP primarily leads to changes in bone tissue, whereas OA affects multiple tissues, including cartilage, subchondral bone, and synovial tissue.

DNA methylation, involving the conversion of cytosine to 5-methylcytosine, primarily occurs at CpG sites—cytosine residues located 5′ to guanosine—within CpG islands in mammals. This modification can either inhibit or facilitate the binding of regulatory proteins to DNA, thereby influencing gene expression. Generally, DNA hypermethylation is associated with gene repression [13]. Currently, a promising area of research is the study of methylation profiles of genes involved in the regulation of chondrogenesis and osteogenesis, as well as the mechanisms of connective tissue component degradation. Of particular interest are the genes Dickkopf WNT Signaling Pathway Inhibitor 1 (*DKK1*), Ras homolog family member J (*RHOJ*), and SRY-Box Transcription Factor 6 (*SOX6*). Although they are implicated in the development of both OA and OP and have been identified as differentially methylated in epigenome-wide studies, their precise contribution at the CpG level remains largely unexplored.

For instance, *DKK1* is a gene encoding a protein that inhibits the Wnt signaling pathway [14], leading to the suppression of canonical Wnt pathway activation and blocking osteoblast proliferation and differentiation. Therefore, it may serve as a potential target for osteoporosis treatment [15]. SOX6, together with SOX5 and SOX9, forms the SOX Trio, which is a critical regulator of chondrogenesis, stimulating the differentiation of mesenchymal stem cells into chondrocytes and subsequent cartilage formation [16]. Osteoarthritic chondrocytes have been found to have reduced concentrations of SOX6 and SOX9 [17]. RHOJ belongs to the Cdc42 subfamily of Rho GTPases and is highly expressed in endothelial cells, playing an important role in regulating angiogenesis in various tissues, including bone and cartilage [18].

The aim of this study was to analyze the methylation levels of CpG sites in the *DKK1*, *RHOJ* and *SOX6* genes among patients with osteoarthritis, osteoporosis, and their combined variant.

## 2. Materials and Methods

### 2.1. Patients

A cross-sectional study was conducted on 96 postmenopausal women, who were divided into three groups: women with a combination of osteoporosis and osteoarthritis (n = 24, mean age 58.23 ± 2.3 years, BMI 25.02 ± 1.8), women with osteoarthritis without osteoporosis (n = 34, mean age 57.02 ± 3.1 years, BMI 28.14 ± 2.2), and a control group without bone pathology (n = 38, mean age 55.2 ± 3.4 years, BMI 27.46 ± 1.4). Written informed consent was obtained from each participant. The study was conducted in accordance with the “Ethical Principles for Medical Research Involving Human Subjects” and was approved by the local ethics committee of the Bashkir State Medical University of the Ministry of Health of Russia. BMD levels were measured using dual-energy X-ray absorptiometry (DEXA) with the Hologic QDR 4500/A DXA system (Hologic, Inc., Marlborough, MA, USA) at standard locations (hip and lumbar spine). The overall sample was divided according to the T-score criterion—from +2.5 to −0.9 standard deviations (SDs) indicated normal BMD, values from −1.0 to −2.5 SD indicated osteopenia, and values below −2.5 indicated osteoporosis (according to the World Health Organization recommendations). The presence of osteoporotic fractures in standard locations (hip axis and lumbar spine) in general and individually, as well as in combination with any other skeletal fractures, was also considered in the patients. The diagnosis of OA was based on the clinical criteria of the American College of Rheumatology with radiological confirmation. Exclusion criteria for all groups were: diabetes mellitus, inflammatory systemic connective tissue diseases, upper limb injuries, acute infectious process, decompensated heart failure, chronic kidney disease with a pronounced decrease in glomerular filtration rate (stage 4–5, mL/min/1.73 m^2^), and occupations or hobbies associated with increased stress on the hand joints. Bone mineral density was assessed in all study participants using dual-energy X-ray absorptiometry at standard sites on a Lunar Prodigy Advance device (GE Healthcare, Waukesha, WI, USA).

### 2.2. DNA Methylation Analysis

DNA was extracted from peripheral blood leukocytes using the QIAamp DNA Blood Kit (QIAGEN, Hilden, Germany). DNA quality and concentration were assessed with a NanoDrop 1000 spectrophotometer (Thermo Scientific, Waltham, MA, USA) and a Qubit 4 fluorometer (Thermo Scientific, USA), respectively. To analyze the methylation profiles of the *DKK1*, *RHOJ*, and *SOX6* genes, genomic DNA was subjected to bisulfite conversion using the EpiTect Fast DNA Bisulfite Kit (QIAGEN, Germany).

Pyrosequencing was subsequently performed on a PyroMark Q24 platform (QIAGEN, Germany). Briefly, bisulfite-converted DNA was amplified by PCR using the PyroMark PCR Kit (QIAGEN, Germany) with specific primers, one of which was biotinylated. The single-stranded DNA template, prepared using the biotin-streptavidin separation system, was then sequenced with a sequencing primer and the PyroMark Gold Q24 Reagents (QIAGEN, Germany). CpG site selection was performed using a two-step strategy combining the a priori biological relevance of genes with technical criteria for quantitative analysis accuracy. In the first step, candidate genes *DKK1*, *SOX6*, and *RHOJ* were selected based on their key role in bone and cartilage homeostasis and their documented involvement in the pathogenesis of osteoarthritis and osteoporosis. Next, to ensure high analytical validity of pyrosequencing measurements, we used commercially available, pre-validated primer sets optimized for the GeneGlobe platform (QIAGEN). This system incorporates rigorous filtering for amplification specificity and the ability to efficiently read CpG positions, which is critical for the reproducibility of quantitative methylation assessments. For each gene, the system offered a limited number of technically approved primer sets, which determined the regions analyzed.

We selected five CpG sites in the *DKK1* gene, one in *RHOJ*, and one in *SOX6*. This selection was based on two factors: their underrepresentation in earlier epigenetic studies of OA and OP, and an optimal primer design generated automatically via the GeneGlobe web interface, using QIAGEN’s technology for CpG island analysis (URL: https://geneglobe.qiagen.com/ru/customize/pyrosequencing/pyromarkcpgandarrayvalidationassays, accessed on 15 October 2025). The specific genomic regions analyzed were: an exon of DKK1 (chr10:52314447–52314480, 5 CpG sites), an upstream regulatory intron of *RHOJ* (chr14:63203974–63203995, 1 CpG site), and an exon of *SOX6* (chr11:15968600–15968612, 1 CpG site). The sequences of the studied regions and primers are provided in Table 1. The characteristics of the analyzed sequences are presented in Table 2.

- IUPAC ambiguity codes: Y = C/T (representing CpG sites where cytosine methylation status is determined); R = A/G
- Genomic coordinates and region characteristics are provided in Table 1
- Pyrosequencing was performed using PyroMark CpG Assays (Qiagen, Hilden, Germany) according to the manufacturer’s protocol
- The dispensation order represents the original genomic sequence; after bisulfite conversion, unmethylated cytosines are converted to uracil (read as thymine), while methylated cytosines remain as cytosine
- All controls performed within the manufacturer’s specified ranges (methylated control: >95% methylation; unmethylated control: <5% methylation).

### 2.3. Statistical Analysis

Statistical analysis was performed using R software (RStudio, version 4.3.1). Before selecting comparative tests, we separately assessed two key assumptions: 1. Normality of distribution in each group was assessed using the Shapiro–Wilk test. 2. Homogeneity of differences between groups was assessed using Levene’s test. Based on these assessments, the following statistical methods were selected: to compare two groups (e.g., pooled cohorts of patients and controls), Student’s *t*-test was used for normally distributed data with equal variances, otherwise, the Mann–Whitney U-test was used. To compare three independent groups, one-way analysis of variance (ANOVA) was used for parametric data with homogeneous variances, followed by Tukey’s post hoc test to obtain significant results; For nonparametric data, the Kruskal–Wallis test with pairwise Mann–Whitney U-tests and Bonferroni correction were used.

To evaluate the prognostic significance of methylation profiles, a multinomial logistic regression model was built, and odds ratios (OR) with 95% confidence intervals were calculated. Model quality was assessed using residual deviance and the Akaike information criterion (AIC). The model’s diagnostic performance was evaluated using receiver operating characteristic (ROC) analysis, with multiclass ROC curves and the area under the curve (AUC) calculated. All analyses were performed using a stratified split of the dataset into training (80%) and testing (20%) subsets. Results were visualized using the ggplot2, pROC, and ggpubr packages (version 1.13).

## 3. Results

### 3.1. Summary Assessment of the Data

**Figure 1:**
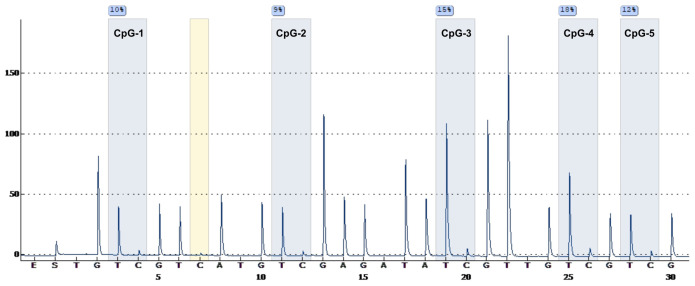
A pyrogram of the methylation profile in the *DKK1* gene. The image is a screenshot from the interface for visualizing pyrosequencing results in PyroMark Q24 Software v2.0.8. The numbering of CpG sites is indicated.

A detailed analysis of methylation patterns in the *DKK1* gene CpG sites (Figure 1) revealed a complex and variable profile across the study groups. For sites *DKK1* CpG1 and *DKK1* CpG2, the control group demonstrated the highest methylation levels with mean values of 58.32% and 53.45% respectively, while the lowest levels were observed in the osteoporosis and osteoarthritis group (38.5% and 45.39%). Interestingly, an inverse pattern emerged at sites *DKK1* CpG4 and *DKK1* CpG5, where the comorbid pathology group showed the highest mean values (54.2% and 50.64%), exceeding those in the control group (46.22% and 46.32%). The aggregate *DKK1* (mean value methylation level) measure was most elevated in the osteoarthritis-only patient group (58.65%), suggesting distinct methylation alterations specific to different clinical disease profiles.

**Figure 2:**
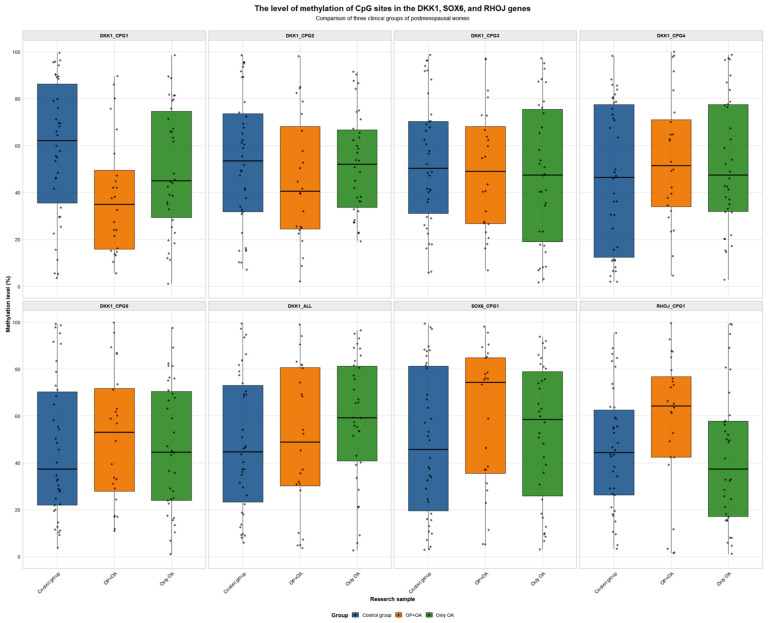
The level of methylation of CpG sites in the *DKK1*, *SOX6*, and *RHOJ* genes in the studied subgroups of women. The visualization of the presented statistics is based on Rstudio (version 4.3.1).

Examination of median values and interquartile ranges confirmed these patterns while providing additional insight into within-group variability. At CPG1 site *DKK1*, the control group median (62.12%) substantially exceeded those in both patient groups (35.01% and 44.97%), with the control group’s interquartile range (35.47–86.25%) indicating considerable value dispersion. Conversely, at CPG5 site *DKK1*, the osteoporosis and osteoarthritis group demonstrated a higher median (53.01%) compared to both the control (37.37%) and osteoarthritis-only groups (44.59%). Particularly noteworthy was site aggregate *DKK1* (mean value methylation level), where the osteoarthritis-only group exhibited not only an elevated median (59.13%) but also a relatively narrow interquartile range (40.83–81.17%), potentially indicating a more homogeneous methylation profile within this patient subgroup. Investigation of *RHOJ* and *SOX6* genes revealed additional features of epigenetic regulation. For the *RHOJ* gene, the osteoporosis and osteoarthritis group displayed substantially higher median methylation (64.27%) compared to both the control group (44.42%) and notably the osteoarthritis-only group (37.37%). A similar, though less pronounced, trend was observed for the *SOX6* gene, where the median in the comorbid pathology group (74.34%) exceeded those in the other groups (45.72% and 58.47%). Collectively, these findings indicate complex and multidirectional alterations in the methylation profiles of the investigated genes, which may reflect distinct molecular mechanisms underlying OP and OA, as well as their comorbid presentation (Figure 2).

### 3.2. Association Analysis

#### 3.2.1. Association Analysis of DKK1 Gene Methylation

**Figure 3:**
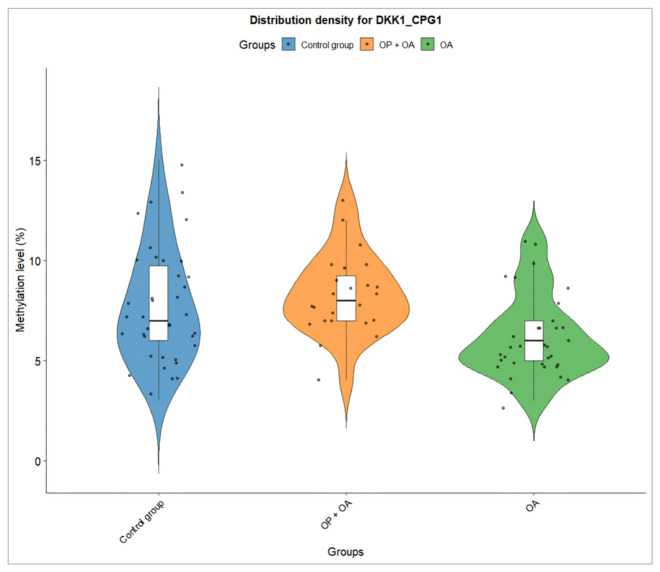
Distribution density, graph for CpG site 1 *DKK1* gene.

**Figure 4:**
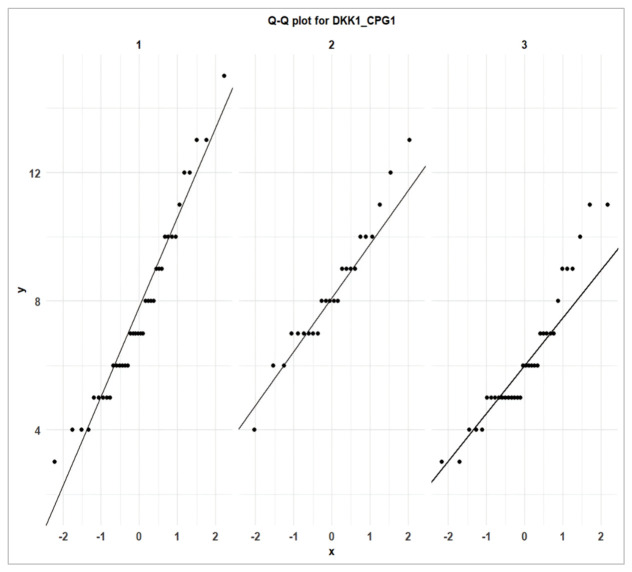
Q−Q plot of methylation values distribution for CpG site 1 *DKK1* gene.

Statistical analysis of the CPG1 site of *DKK1* gene was conducted to assess differences in methylation levels between the study groups. Distribution density graph for *DKK1* CpG site presented in Figure 3 and Figure 4. The Shapiro–Wilk test indicated that the assumption of normality was met for the control group (*p* = 0.099) and the OP+OA group (*p* = 0.473), but was violated for the OA without OP group (*p* = 0.007). Consequently, an initial independent samples *t*-test comparing the combined patient groups (OP+OA and OA without OP) to the control group revealed no statistically significant difference (t = 1.1279, df = 66.815, *p* = 0.2634). However, the non-parametric Kruskal–Wallis test, applied for the comparison of all three groups, identified a highly significant overall difference (χ^2^ = 13.56, df = 2, *p* = 0.001136). Post-hoc pairwise comparisons using the Mann–Whitney U test with Bonferroni correction were performed to delineate these differences. The analysis revealed no statistically significant difference in methylation levels between the Control group and the OP+OA group (p_adj_ = 0.540), nor between the Control group and the OA without OP group (p_adj_ = 0.056). In contrast, a statistically significant difference was observed between the two patient groups, with the OP+OA group demonstrating a significantly higher level of methylation compared to the OA without OP group (p_adj_ = 0.00067).

In summary, while methylation at the CPG1 site of *DKK1* gene did not distinguish the patient cohorts from the healthy controls, it revealed a nominally significant epigenetic discrepancy. between the two clinical phenotypes, with the comorbid osteoporosis and osteoarthritis group exhibiting elevated methylation relative to the group with OA alone.

**Figure 5:**
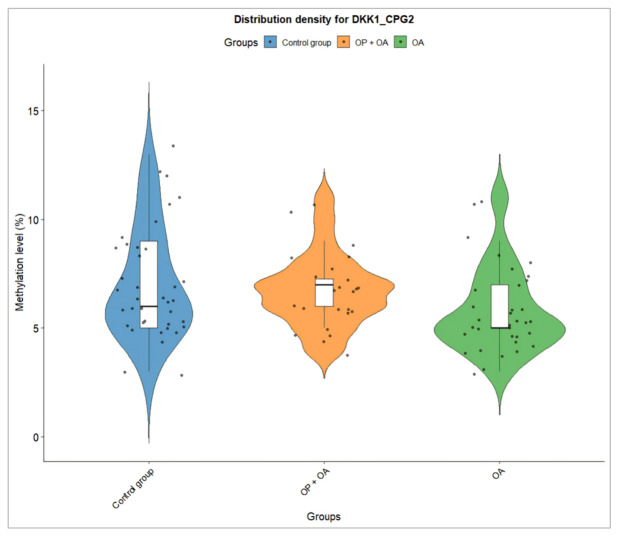
Distribution density graph for CpG site 2 *DKK1* gene.

**Figure 6:**
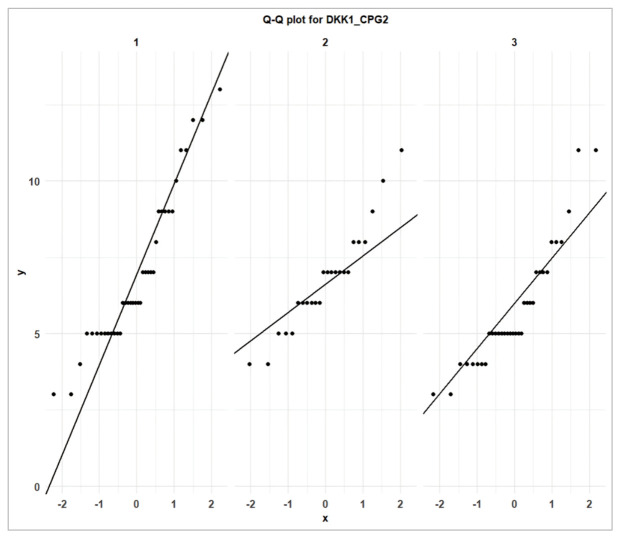
Q−Q plot of methylation values distribution for CpG site 2 *DKK1* gene.

Analysis of the CPG2 site of *DKK1* gene methylation patterns revealed distinct statistical characteristics. Distribution density graph for *DKK1* CpG site 2 presented in Figure 5 and Figure 6. The Shapiro–Wilk test indicated non-normal distribution for the Control group (*p* = 0.006), the OA without OP group (*p* = 0.002), and the OP+OA group (*p* = 0.159), necessitating non-parametric statistical approaches. Initial comparison between the Control group and combined patient cohorts using the Mann–Whitney U test showed no significant difference (W = 1281, *p* = 0.1744). However, the Kruskal–Wallis test comparing all three groups independently demonstrated a statistically significant overall difference in methylation levels (χ^2^ = 6.944, df = 2, *p* = 0.031).

Post-hoc pairwise analysis with Bonferroni correction revealed no statistically significant differences between any specific group pairs. The comparison between Control and OP+OA groups showed no significant difference (p_adj_ = 0.478), nor did the comparison between Control and OA without OP groups (p_adj_ = 0.077). Similarly, the comparison between the two patient groups (OP+OA versus OA without OP) failed to reach statistical significance after correction (p_adj_ = 0.065). Despite these non-significant pairwise results, the observed median values indicated a consistent pattern: the OP+OA group demonstrated the highest methylation level (median = 7), followed by the Control group (median = 6), with the OA without OP group showing the lowest methylation (median 5).

In summary, while the overall analysis of CPG1 site of *DKK1* gene methylation revealed significant variation among the three groups, post-hoc testing did not identify statistically significant differences between any specific group pairs after multiple comparison correction. The consistent directional pattern observed across all comparisons, with the comorbid OP+OA group consistently exhibiting the highest methylation levels, suggests a potential biological trend that warrants further investigation in larger cohorts.

The analysis of methylation patterns at the *DKK1* CPG3 and *DKK1* CPG4 loci did not yield statistically significant associations with the patient groups.

**Figure 7:**
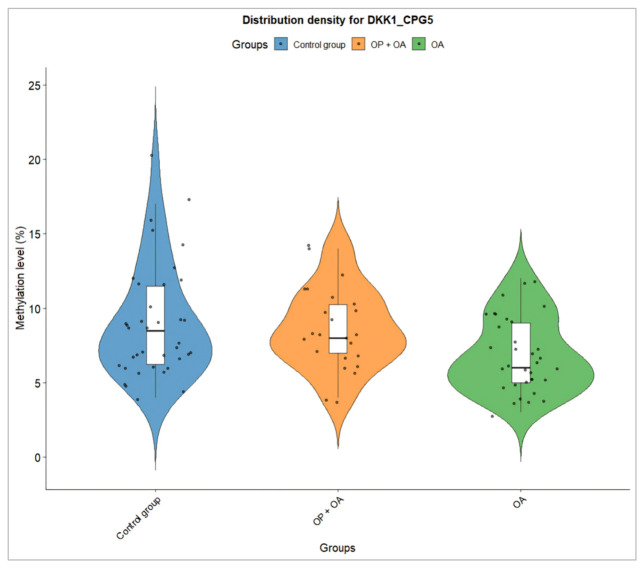
Distribution density graph for CpG site 5 *DKK1* gene.

**Figure 8:**
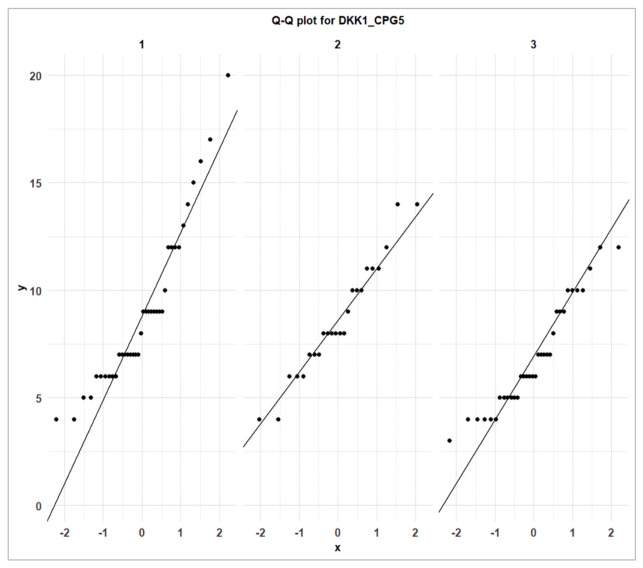
Q−Q plot of methylation values distribution for CpG site 5 *DKK1* gene.

Statistical analysis of CPG5 site of *DKK1* gene methylation levels revealed a non-normal distribution pattern across the study groups, as confirmed by Shapiro–Wilk tests (control group: W = 0.002; OP+OA group: W = 0.392; OA-only group: W = 0.027). Distribution density graph for *DKK1* CpG site 2 presented in Figure 7 and Figure 8. The initial comparison between the control group and combined patient groups using the Mann–Whitney test showed no statistically significant difference (W = 1321, *p* = 0.099). However, the Kruskal–Wallis test examining all three groups independently demonstrated significant overall differences in methylation levels (χ^2^ = 8.606, df = 2, *p* = 0.014).

Post-hoc pairwise comparisons with Bonferroni correction identified specific intergroup differences. No significant methylation difference was observed between the control and OP+OA groups (adjusted *p* = 0.774), despite the control group showing a marginally higher median value (8.5% versus 8.0%). In contrast, statistically significant differences emerged between the control and OA-only groups (p_adj_ = 0.031), with the control group maintaining higher methylation levels (median 8.5% versus 6.0%). Similarly, a significant difference was detected between the OP+OA and OA-only groups (adjusted *p* = 0.042), where the OP+OA group demonstrated elevated methylation relative to the OA-only group (median 8.0% versus 6.0%). These results indicate a complex methylation landscape at the CPG5 site of *DKK1* gene, where the OA-only group consistently exhibits the lowest methylation levels, while both the control and OP+OA groups maintain comparatively higher methylation, though not significantly different from each other. The findings suggest that osteoarthritic pathology without comorbid osteoporosis is associated with distinct epigenetic modifications at this specific CpG site.

**Figure 9:**
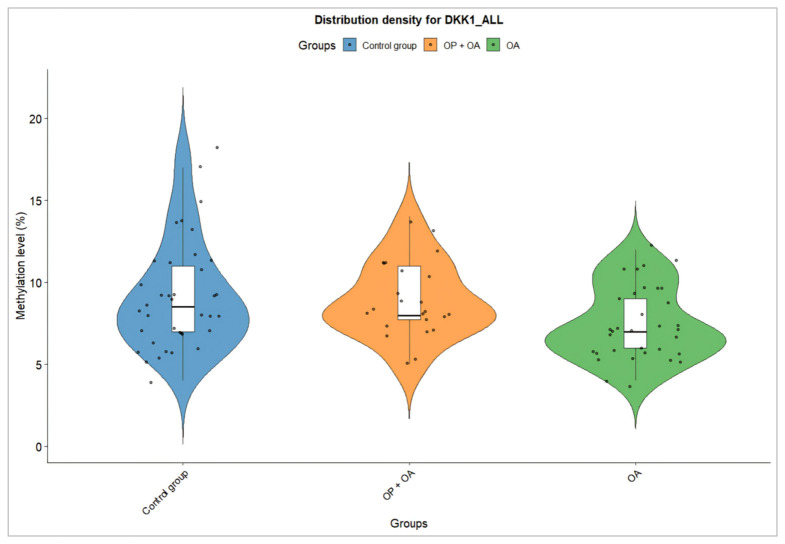
Distribution density graph for CpG 1–5 sites average *DKK1* gene.

**Figure 10:**
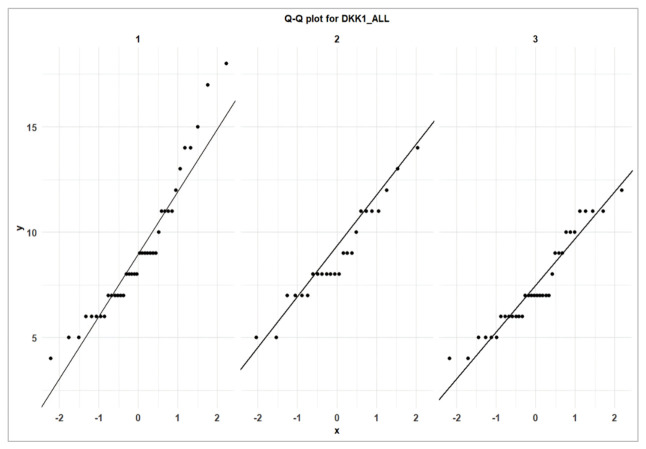
Q−Q plot of methylation values distribution for CpG 1–5 sites average *DKK1* gene.

Analysis of the composite methylation score, derived from averaging the values of the five individual *DKK1* CpG sites (DKK1_CPG 1–5), revealed a distinct pattern. Distribution density graph presented in Figure 9 and Figure 10. The data followed a non-normal distribution, as indicated by Shapiro–Wilk tests (Control: *p* = 0.009; OP+OA: *p* = 0.204; OA-only: *p* = 0.018). While an overall Kruskal–Wallis test comparing all three groups showed a nominally significant difference (χ^2^ = 7.13, df = 2, *p* = 0.028), a more nuanced picture emerged from post-hoc pairwise comparisons with Bonferroni correction. These analyses revealed that the initial global significance was driven by a specific and statistically significant difference between the OP+OA group and the OA-only group (adjusted *p* = 0.042), with the former exhibiting a higher median methylation level (8% vs. 7%).

In contrast, no statistically significant differences were found between the control group and the OP+OA group (p_adj_ = 0.463) or between the control and OA-only groups (adjusted *p* = 0.095), despite the control group showing a marginally higher median value (8.5%). This pattern suggests that the averaged *DKK1* methylation profile is most effective in distinguishing between the two patient phenotypes, rather than separating the control group from the diseased cohorts.

#### 3.2.2. Association Analysis of *RHOJ* and *SOX6* Genes Methylation

In contrast to the site-specific associations observed in the *DKK1* gene, the analysis of the *RHOJ* and *SOX6* genes did not reveal statistically significant differences in methylation levels after correction for multiple comparisons (Table 3). For the CPG1 site of *RHOJ* gene, a one-way ANOVA comparing all three groups showed no significant overall effect (*p* = 0.090). Subsequent post-hoc Tukey tests with Bonferroni correction confirmed the absence of significant pairwise differences between any of the groups (all adjusted *p* > 0.17), despite observable numerical differences in medians. Similarly, for the CPG1 site of *SOX6* gene, while the initial Mann–Whitney test comparing the control group to the combined patient groups suggested a potential difference (*p* = 0.019), the more rigorous Kruskal–Wallis test across all three groups was not significant (*p* = 0.063).

Most importantly, the post-hoc pairwise comparisons with Bonferroni correction failed to identify any significant differences between the specific groups (all adjusted *p* > 0.09). These results indicate that, within the statistical power of this study, methylation levels at the investigated CpG sites of the *RHOJ* and *SOX6* genes are not associated with the distinct clinical phenotypes of osteoporosis and osteoarthritis.

#### 3.2.3. Development of a CpG-Site Based Classification Model for Clinical Phenotypes

To comprehensively evaluate the diagnostic potential of the methylation signature, a multinomial logistic regression model was developed using the most informative CpG sites identified in the univariate analysis. The final model incorporated four key predictors: *DKK1*_CPG1, *DKK1*_CPG2, *DKK1*_CPG3, *DKK1*_CPG4, *DKK1*_CPG5, *SOX6*_CPG1, *RHOJ*_CPG1 (Table 4).

**Figure 11:**
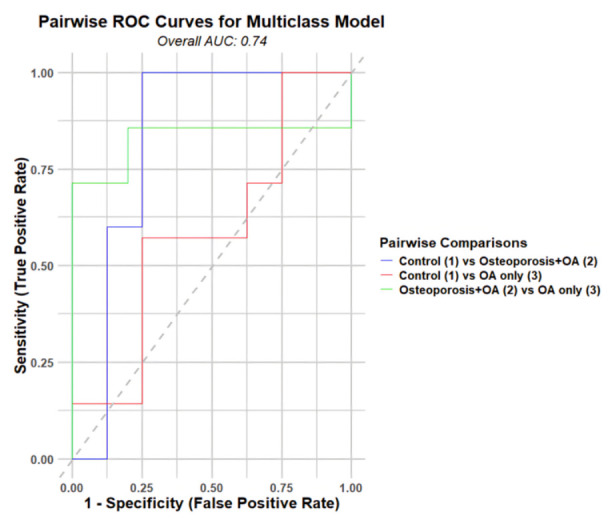
Prognostic multinomial model including *DKK1*_CPG1, *DKK1*_CPG2, *DKK1*_CPG3, *DKK1*_CPG4, *DKK1*_CPG5, *SOX6*_CPG1, *RHOJ*_CPG1 predictors.

The model was trained and validated using a stratified 80/20 data split to ensure robust performance estimation. The overall diagnostic performance, assessed through multiclass Receiver Operating Characteristic (ROC) analysis, demonstrated substantial discriminatory power with an aggregate Area Under the Curve (AUC) of 0.74 (Figure 11). This indicates that the integrated methylation profile of these four CpG sites possesses good classification accuracy for distinguishing between the three clinical phenotypes. The pairwise ROC comparisons further refined this assessment, revealing the model’s specific ability to differentiate between each distinct group pairing: control versus osteoporosis+OA, control versus OA only, and osteoporosis+OA versus OA only.

The strong performance of this multivariate model, which combines sites from both the *DKK1* and *SOX6* genes, suggests synergistic effects and underscores the advantage of using composite epigenetic profiles over single-site analyses for classifying complex musculoskeletal disorders. This approach provides a more nuanced understanding of the collective contribution of these epigenetic markers to disease pathophysiology and highlights their potential as a multi-dimensional biomarker panel.

**Figure 12:**
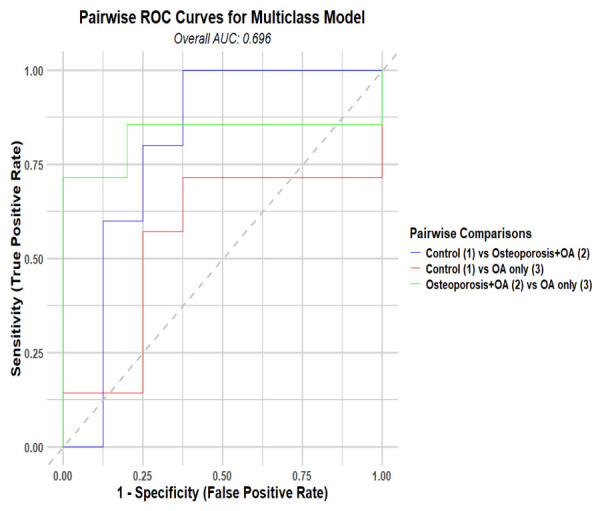
Prognostic multinomial model including *DKK1*_CPG1, *DKK1*_CPG2, *DKK1*_CPG5, and *SOX6*_CPG1.

Building upon the initial findings, we further refined the predictive model through a feature selection process to optimize its discriminatory power. The final, optimized multinomial logistic regression model retained four key CpG sites: *DKK1*_CPG1, *DKK1*_CPG2, *DKK1*_CPG5, and *SOX6*_CPG1 (Table 5). This refined model revealed a more nuanced architecture of epigenetic associations than previously observed. Specifically, for discriminating the combined osteoporosis+OA phenotype from the Control group, *DKK1*_CPG1 emerged as a strong positive predictor (coefficient = 1.87, *p* = 0.0003), while *DKK1*_CPG2 served as a significant negative predictor (coefficient = −1.57, *p* = 0.0077). In contrast, for distinguishing the OA-only phenotype from Controls, a different pattern was observed, where *DKK1*_CPG5 was the most significant negative predictor (coefficient = −0.41, *p* = 0.0102). The *SOX6*_CPG1 site contributed significantly to both comparative frameworks (*p* = 0.0463 and *p* = 0.0479, respectively). The model’s overall diagnostic performance, while slightly attenuated from initial estimates, maintained average discriminatory power with a multiclass AUC of 0.696 (Figure 12). This refined analysis confirms that the combinatorial methylation signature of *DKK1* and *SOX6* genes provides optimal framework for patient stratification, while highlighting that distinct CpG sites drive the classification for different clinical presentations. The feature selection process thus successfully identified a parsimonious yet potential informative set of epigenetic markers for differentiating complex musculoskeletal disease phenotypes.

### 3.3. Functional Interpretation of Differential Methylation of Studied Genes

Our analysis identifies distinct methylation patterns in two genes—*DKK1* and *SOX6*—across osteoarthritis (OA), osteoporosis (OP), and comorbid OA+OP cohorts, implicating their roles in diseases-specific epigenetic dysregulation.

*DKK1*: This gene encodes a secreted inhibitor of the Wnt/β-catenin signaling pathway, a master regulator of osteoblastogenesis and bone homeostasis. We observed significant hypermethylation at specific CpG sites within its exon (positions 52314447–52314480) in the comorbid OA+OP group compared to isolated OA. Conversely, hypomethylation at another CpG site was associated with isolated OA versus controls. Given that exon methylation can influence mRNA splicing, stability, and translation efficiency, we hypothesize that the observed hypermethylation in the comorbid state may represent a mechanism to transcriptionally dampen the synthesis of the *DKK1* protein, whose overexpression is known to suppress bone formation. The hypomethylation in isolated osteoarthritis could facilitate increased *DKK1* activity, contributing to aberrant subchondral bone remodeling, but this requires confirmation.

*SOX6*: A transcription factor critical for chondrocyte differentiation and cartilage integrity, *SOX6* prevents chondrocyte hypertrophy and maintains the extracellular matrix. Our model identifies a CpG site within its exon (15968600–15968612) as a significant predictor. Methylation in this exon could directly interfere with the translation of a functional protein domain or mRNA stability. We hypothesize that methylation-driven suppression of *SOX6* expression contributes to the loss of the stable chondrocyte phenotype, promoting cartilage degradation in osteoarthritis.

*RHOJ*: This gene, selected based on prior genome-wide significance, encodes a GTPase involved in angiogenesis and cytoskeletal organization. The CpG site of interest is located in a regulatory upstream intron (63203974–6320399). Methylation in such intronic regions can profoundly alter enhancer activity and transcription factor binding. However, our study did not confirm the significance of the studied CpG site, requiring a focus on other differentially methylated regions of the gene.

## 4. Discussion

Our investigation reveals a complex epigenetic landscape underpinning Osteoarthritis (OA) and Osteoporosis (OP). The most significant finding is the pronounced hypermethylation of the DKK1 exon observed in patients with comorbid OA and OP. This initially appears counterintuitive, as DKK1 is a well-established negative regulator of bone formation, and its overexpression is a known feature of OP. However, we hypothesize the involvement of a compensatory epigenetic mechanism. This assumption currently remains theoretical and necessitates stringent functional confirmation. We propose that the chronic overexpression of DKK1, driven by the combined inflammatory and metabolic stress of both diseases, may eventually initiate a negative feedback loop. In response, the epigenetic machinery could increase methylation in specific exonic regions, thereby attempting to post-transcriptionally limit the production of this potentially deleterious protein. This interpretation aligns with broader evidence in rheumatology, where disease-specific DNA methylomes demonstrate active epigenetic remodeling in response to a pathological microenvironment. Consequently, DKK1 hypermethylation likely signifies a state of severe, systemic joint failure, where compensatory mechanisms are actively engaged.

The simultaneous dysregulation of SOX6 and DKK1 further underscores the multi-factorial nature of the disease, affecting both cartilage and synovial tissues. The observed hypomethylation of DKK1 in isolated OA, which would be expected to increase its activity, is consistent with its proposed pathogenic role in subchondral bone sclerosis. Furthermore, the inclusion of the SOX6 exon in our predictive model highlights the critical role of chondrocyte loss.

In recent years, epigenetic factors have gained significant recognition as crucial biomarkers for aging and age-related diseases, holding substantial clinical relevance for diagnosis, prognosis, and predicting therapeutic response. Among age-related bone metabolism disorders, OP and OA are the most prevalent. These are complex, multifactorial, late-onset disorders with a strong epigenetic component that differentially affect bone [19,20], leading to an imbalance between osteoblast-mediated bone formation and osteoclast-mediated bone resorption. Recent studies examining global and gene-specific methylation profiles in whole blood have identified epigenetic signatures broadly associated with the progression of OP and OA, potentially serving as non-invasive prognostic biomarkers. However, it is important to note that the tissues primarily involved in the pathogenesis of these two diseases are distinct: OP predominantly leads to changes in bone tissue, whereas OA impacts multiple tissues, including cartilage, subchondral bone, and synovial tissue.

DNA methylation represents a crucial epigenetic mechanism, involving heritable modifications to gene activity or function without altering the underlying DNA sequence. This process plays a pivotal role in regulating gene expression through diverse molecular pathways.

DNA methylation occurs in distinct genomic contexts, including intergenic regions, CpG islands, and gene bodies. In intergenic regions, methylation typically silences mobile and viral elements, thereby preventing their replication and integration, which could otherwise lead to gene disruption and DNA mutations. This mechanism suppresses the expression of potentially harmful genetic elements. CpG islands, which comprise about 1–2% of the entire genome and are rich in GC sites, are frequently located in gene promoters and 5′-regulatory regions [21,22]. Here, DNA methylation can regulate gene expression by either recruiting proteins involved in gene repression or by physically impeding the binding of transcription factors to DNA. While the exact mechanisms of gene body methylation in regulating gene expression are still under investigation, transcribed regions can exhibit extensive methylation, often showing a positive correlation with gene expression levels. Methylation in these areas may contribute to maintaining transcriptional efficiency by silencing alternative promoters, retrotransposon elements, and other functional elements [23].

Recent epigenetic studies have shed light on the role of DNA methylation in Osteoporosis (OP). An analysis conducted in a cohort of Chinese postmenopausal women (n = 30 OP patients vs. n = 30 healthy subjects) revealed reduced methylation within a CpG island in the promoter of the TBC1F8 gene in OP patients. This finding suggests a novel avenue for investigating the functional role of this gene region in OP pathogenesis [24]. Further insights come from analyzing DNA methylation in a CpG island within the promoter region of the BMP2 gene, a crucial bone factor implicated in OP pathogenesis. In a whole blood analysis of Asian-Indian individuals, the frequency of methylated cytosines across 14 CpG sites in this promoter region was significantly higher in OP patients (0.7, n = 24) compared to healthy controls (0.25, n = 24). This epigenetic regulatory mechanism was corroborated by lower BMP2 expression levels and transcriptional activity in OP patients, suggesting it could alter downstream genes in the BMP2 signaling pathway, thereby impacting osteoblastogenesis and bone formation [25].

Further research has explored the epigenetic regulation of the sclerostin (SOST) gene, a key inhibitor of bone formation. One study observed that increased methylation of the SOST gene in patients with low Bone Mineral Density (BMD) correlated with reduced SOST mRNA levels in bone and lower serum sclerostin levels [26]. This aligns with prior work by Delgado-Calle’s team [27], which reported hypomethylation in the SOST gene promoter region in human osteocytes, indicative of active sclerostin production. The observed hypermethylation in OP patients is thus hypothesized to be a compensatory mechanism: by decreasing serum sclerostin, it could alleviate the inhibition of Wnt signaling and consequently promote bone formation [28]. This compensatory role was further supported by a study involving 12 postmenopausal women with OP and bone fractures and 8 healthy volunteers, which confirmed elevated methylation levels in two CpG islands within the SOST gene promoter. Bisulfite sequencing revealed a hypermethylation pattern in bone tissue from both osteoporotic fracture groups and non-osteoporotic fracture groups [29]. These findings collectively highlight the complex and not yet fully understood role of DNA methylation in SOST regulation within OP pathogenesis, underscoring the need for more comprehensive investigations into its contribution to bone metabolism modulation.

In 2017, Del Real and colleagues investigated DNA methylation profiles during osteogenic induction of mesenchymal stem cells obtained from patients with hip fractures and osteoarthritis. Their research demonstrated an accelerated demethylation process in genes critical for osteoblast differentiation, leading to increased expression of osteogenic differentiation inducer genes such as *RUNX2* or *OSX* [30]. Moreover, mechanical loading on bone is known to elevate the expression of the BGP gene (bone gamma-carboxyglutamate (Gla) protein), thereby promoting osteoblast differentiation [31].

The Receptor Activator of Nuclear Factor kappa-B Ligand (RANKL) and Osteoprotegerin (OPG) system is crucial for bone remodeling. RANKL interacts with its receptor, RANK, to stimulate osteoclast formation and survival, while OPG acts as a decoy receptor, antagonizing RANKL and preventing excessive bone resorption. DNA methylation has been shown to reduce the transcriptional levels of both RANKL and OPG genes, a role confirmed by the use of specific demethylating agents [27,32]. Elucidating the precise transcriptional regulation mechanisms of the RANKL-OPG system genes holds significant promise for OP therapy. OPG expression is promoted by various factors, including fibroblast growth factor 21, PGE2, transforming growth factor beta, and estrogen, while RANKL expression is regulated by agents such as PAPSS2, IL-3, tumor necrosis factor alpha, and Wnt ligands. A study by Peng Wang et al. [33] examined the methylation profile and expression levels of OPG and RANKL in femoral bone tissue from patients with osteoporotic and non-osteoporotic fractures. They found that RANKL gene expression was significantly higher in the osteoporotic fracture group, coinciding with significantly lower CpG methylation in its promoter. Conversely, OPG showed lower gene expression levels and a higher degree of methylation in the fracture patient group [33].

A large-scale study conducted by Yu Zhou on postmenopausal women with osteoporosis identified 13 differentially methylated sites (*p* < 0.05). Hypermethylated genes included PLEKHA2, PLEKHB1 (encoding pleckstrin homology domains A2 and B1), PNPLA7 (encoding patatin-like phospholipase domain 7), SOX6 (transcription factor), SYK (non-receptor tyrosine kinase), GNA11 (G protein subunit alpha 11), and PRKCZ (protein kinase C zeta type). Conversely, hypomethylated genes comprised SCD (angiotensin-converting enzyme), MGST3 (microsomal glutathione S-transferase), TGFB3, TGFB5 (transforming growth factors beta 3 and 5), COL4A1 (collagen type IV alpha-1), and TSNAX (translin-associated factor X) [34].

Regarding Osteoarthritis (OA), four specific susceptibility genes—insulin-like growth factor binding protein 7 (IGFBP7), low-density lipoprotein receptor-related protein 5 (LRP5), FTO, and nuclear receptor corepressor 2 (NCOR2)—have been identified that display consistent methylation patterns in both subchondral bone and cartilage. This suggests shared epigenetic mechanisms operating in both the subchondral bone and the eroded cartilage of OA patients [35].

In contrast, Zhang et al. [36] examined the subchondral bone of knee joints from 12 individuals at various disease stages, aiming to characterize OA progression in relation to epigenetic changes. Their findings revealed distinct methylation patterns in cartilage compared to subchondral bone, indicating a tissue-specific role for DNA methylation during the course of OA.

Beyond separate investigations into epigenetic factors in Osteoporosis (OP) and OA, several researchers have posited a common genetic/epigenetic basis for both diseases, suggesting overlapping pathogenetic mechanisms [37]. Delgado-Calle and colleagues pioneered research into the role of DNA methylation in human bone from OP and OA patients, analyzing two crucial bone metabolism regulators: RANKL and its soluble decoy receptor OPG. Despite observing significant differences in gene expression between the two pathological groups, they did not identify any variations in the RANKL methylation pattern.

More recently, Li et al. conducted an analysis of cancellous bone samples from 12 individuals categorized into four groups: OP, OA, OP and OA co-occurrence (OP+OA), and healthy controls. This study unveiled increased methylation levels across a total of 1222 distinct sites in both the OP and OA groups when compared to the OP+OA group. Furthermore, four genes (*PPIL3*, *NGG1*, *NIF3L1*, *CALHM2*) were commonly identified in both the OP and OA groups [38]. These shared genes may play a role in the initiation and progression of both OP and OA, thereby representing a potential molecular link between these two bone pathologies. Consequently, the investigation of common epigenetic regulatory mechanisms could significantly advance the diagnosis and treatment of bone metabolism disorders.

Interestingly, in chicken embryo chondrocytes, the COL2A1 gene exhibits reduced methylation compared to fibroblasts, a status that remains unchanged even upon chondrocyte dedifferentiation [39]. Similarly, in human articular chondrocytes and mesenchymal stem cells (MSCs) undergoing chondrogenesis, all 74 CpG sites within the promoter region of the COL2A1 gene around its transcription start site are unmethylated [40].

The expression of matrix metalloproteinases (MMPs) is typically low in healthy cartilage but significantly elevated in osteoarthritis (OA), contributing to the degradation of the extracellular matrix. Analysis of the promoter regions for MMP3, MMP9, and MMP13 in OA patients reveals a substantial loss of methylation compared to control groups [41]. However, this demethylation is not uniform across all CpG sites; specific sites show more pronounced changes: −635 bp for MMP3, −36 bp for MMP9, and −110 bp for MMP13 [41].

While ADAMTS5 is considered the primary aggrecanase in OA, ADAMTS4 also plays a role in aggrecan degradation. Demethylation is observed at CpG sites within the ADAMTS4 promoter, with the −753 bp site being the most consistently demethylated region. This is accompanied by a dramatic 700-fold increase in ADAMTS4 expression in the superficial zone of OA cartilage [41,42]. This finding is particularly noteworthy as it suggests that methylation of a single CpG site might be sufficient to influence gene expression, a departure from the earlier belief that widespread CpG methylation was necessary for gene suppression.

Growth factors and their antagonists can also be regulated through varying methylation levels. Bone morphogenetic protein 7 (BMP7), also known as osteogenic protein 1 (OP-1) and a member of the TGF-β superfamily, is crucial for cartilage maintenance and repair. It regulates genes involved in extracellular matrix production, anabolic pathways, bone formation, as well as those controlling cytokines and various catabolic pathways responsible for extracellular matrix degradation and apoptosis [43]. In aged chondrocytes, a positive correlation exists between age and BMP7 promoter methylation, leading to a concomitant decrease in the expression of BMP7 and its regulated genes, such including IGF-1, the IGF-1 receptor (IGF-1R), and ACAN [44]. This age-related increase in BMP7 promoter methylation may contribute to the cartilage loss seen during aging and OA progression.

SOST (sclerostin), a BMP antagonist that modulates mitogenic activity by sequestering BMPs [45], promotes subchondral bone sclerosis and impedes cartilage degradation in OA [46]. In OA chondrocytes, the CpG region of the SOST promoter is hypomethylated, resulting in increased gene expression compared to normal chondrocytes [47].

Growth differentiation factor 5 (GDF5), another member of the TGF-β superfamily, is involved in chondrogenesis and chondrocyte proliferation [48]. A single nucleotide polymorphism (SNP) at rs143383 (C/T) in the 5′ untranslated region of the GDF5 gene is associated with OA and has a functional impact [49]. Demethylation of GDF5, correlating with its increased expression, has been observed in cell lines and joint tissues [50].

Recent research has yielded compelling insights into epigenetic regulatory mechanisms, not only in whole blood but also within key tissues affected by osteoarthritis (OA), such as cartilage, subchondral bone, and synovial membrane [51]. The analysis of extracellular genomic material—including miRNA, lncRNA, sncRNA, mRNA, and cell-free DNA—found in the body fluids of OA patients has revealed aberrantly expressed factors that show promise as novel disease biomarkers [52].

Furthermore, DNA methylation patterns in blood present a significant opportunity for developing non-invasive biomarkers, as they influence the expression of genes implicated in OA pathogenesis. A pioneering pilot study, for instance, is exploring whether an epigenetic signature in blood can predict the future radiographic progression of knee osteoarthritis [53].

Another promising therapeutic avenue lies in gene therapy, specifically through modifying methylation levels in target genes. The RANKL-RANK-OPG system, along with other critical genes involved in connective tissue metabolism, could serve as therapeutic targets for both osteoporosis and OA. To advance this, a comprehensive understanding of how methylation influences the genes within this system is essential.

Consequently, investigating the epigenetic mechanisms crucial for bone and cartilage remodeling represents a highly promising direction for improving the diagnosis and treatment of osteoporosis and osteoarthritis.

## 5. Conclusions

Our data implicate site-specific methylation of *DKK1* and *SOX6* as a molecular correlate of comorbid osteoarthritis and osteoporosis. This targeted epigenetic signature offers a new perspective for understanding the shared etiology of these diseases. However, all the obtained results require replication on an independent cohort of patients, and our assumptions about the mechanisms of the influence of differential methylation on the pathogenesis of osteoporosis and osteoarthritis are theoretical and controversial in nature, requiring additional functional validation.

## Figures and Tables

**Table 1:** Characteristics of the studied genomic regions for pyrosequencing.

Gene	Chromosome	Genome Position	Type of Region	Number of CpG-Sites	Amplicon Length
*DKK1*	10	52314447–52314480	Exon	5	161
*RHOJ*	14	63203974–63203995	Intron	1	268
*SOX6*	11	15968600–15968612	Exon	1	300

**Table 2:** The characteristics of the analyzed sequences.

Gene	Nucleotide Dispensation Order	Analyzed DNA Sequence After Bisulfite Conversion
*DKK1*	TGTCGTCATGTCGAGATATCGTTGTCGTCG	GGYGTAGYGGGAGTTATTYGGGTTTTTGTYGYGA
*RHOJ*	TGTCGATTATGATGA	GYGATTTTTTTTAGAAATAAAGAA
*SOX6*	ATTCGTAGAGAT	TTTYGAAGAAGTAGT

**Table 3:** Methylation levels of *RHOJ* and *SOX6* genes across study groups (mean ± SD).

Gene	Control (n = 38)	OP+OA (n = 24)	OA Only (n = 34)
*SOX6*_CPG1	90.1 ± 2.8	91.3 ± 3.8	90.4 ± 2.4
*RHOJ*_CPG1	54.6 ± 13.2	50.8 ± 14.7	47.6 ± 14.1

**Table 4:** Parameters of statistical significance of the predictive model based on predictors *DKK1*_CPG1, *DKK1*_CPG2, *DKK1*_*CPG3*, *DKK1*_CPG4, *DKK1*_CPG5, *SOX6*_CPG1, *RHOJ*_CPG1.

Predictor	Group 2 Coefficient	Group 2 Z-Value	Group 2 *p*-Value	Group 3 Coefficient	Group 3 Z-Value	Group 3 *p*-Value
(Intercept)	−25.89987	−1.753	0.0796	−9.81274	−0.793	0.4278
*DKK1*_CPG1	1.9080567	3.591	0.0003	0.1377179	0.380	0.7036
*DKK1*_CPG2	−1.60082089	−2.679	0.0074	0.02889876	0.100	0.9206
*DKK1*_CPG3	−0.01794895	−0.050	0.9601	0.03298561	0.147	0.8795
*DKK1*_CPG4	−0.09982607	−0.197	0.8438	0.40648649	1.000	0.3173
*DKK1*_CPG5	−0.2127026	−0.356	0.7220	−0.9140098	−1.922	0.0547
*SOX6*_CPG1	0.2831900	1.791	0.0732	−0.1375688	−1.042	0.3036
*RHOJ*_CPG1	−0.01924619	−0.752	0.4526	−0.03444571	−1.608	0.1078

**Table 5:** Parameters of statistical significance of the predictive model based on predictors *DKK1*_CPG1, *DKK1*_CPG2, *DKK1*_CPG5, and *SOX6*_CPG1.

Predictor	Group 2 Coefficient	Group 2 Z-Value	Group 2 *p*-Value	Group 3 Coefficient	Group 3 Z-Value	Group 3 *p*-Value
(Intercept)	−28.76422	−2.007	0.0447	−10.76648	−0.942	0.0360
*DKK1*_CPG1	1.872434	3.629	0.0003	0.1054602	0.301	0.7632
*DKK1*_CPG2	−1.5711587	−2.669	0.0077	0.1098609	0.361	0.7177
*DKK1*_CPG5	−0.3166884	−1.030	0.3033	−0.4143749	−1.638	0.0102
*SOX6*_CPG1	0.2987700	1.925	0.0463	0.1387771	1.106	0.0479

## Data Availability

The data presented in this study are available on request from the corresponding author.

## Abbreviations

The following abbreviations are used in this manuscript:

OP	Osteoporosis
OA	Osteoarthritis
BMD	Bone mineral density
*DKK1*	Dickkopf WNT Signaling Pathway Inhibitor 1
*RHOJ*	Ras homolog family member J
SOX6	SRY-Box Transcription Factor 6
*SOST*	Sclerostin
MMP	Metalloproteinase
